# Injuries of the Head

**Published:** 1829-01

**Authors:** 


### HOSPITAL REPORTS,

(*Principally condensed from various Periodical Publications.*)

---

### INJURIES OF THE HEAD.

### I. *Violent Concussion, with arterial Bleeding from the Ear.*

J. S., aged forty, a servant, admitted at St. Thomas's Hospital, under the care of Mr. Travers.

October 12th, he fell from a horse upon his head, about noon. At one o'clock he was brought to the hospital, with symptoms of concussion, viz. insensibility, contraction of the pupils, vomiting, a weak pulse at 104, strabismus, and stertorous breathing. There was considerable arterial hemorrhage from the right ear and nose. He had been bled immediately after the accident. The head was shaved, and carefully examined, but no fracture was discovered.

Spirit wash was applied to the scalp.

At two P.M. he was still insensible, passing his stools involuntarily, and vomiting occasionally, which action caused the blood to flow afresh from his ear. The strabismus and stertorous breathing were no longer observable. The pulse had fallen to eighty-four, and continued weak.

Six P.M.—Reaction taking place: pulse ninety-four, and fuller; slight sensibility, great restlessness; another involuntary motion; matter vomited slightly tinged with blood. From this time to nine P.M. the pulse became gradually fuller. At ten P.M. he was ordered

V.S. ad ℥x. Hirudines xx. occipiti. Emp. Lyttæ nuchæ. Contr Lot. spirituosa.

13th.—Has been restless since five o'clock, vomiting frequently. Complains of pain in the forehead and occiput; pulse eighty, and soft; pupils contracted; twitching of the mouth and limbs when asleep.

Pulv. Scam. c. Cal. gr. x.—C. C. occipiti ad ℥xij.—Ol. Ricini p. r. n.

14th.—Slept well until three o'clock; since which has been very restless; answers incoherently; says he is blind; pupils contracted, and quite insensible to light; three stools; pulse eighty, and fuller.

V.S. e venâ jugulari ad ℥xiv. Contr Lot. spirituosa.

Pulse rendered softer and a little quicker by the bleeding.

15th.—Was very restless during a part of the night. Passes his water freely, but has had no motion since yesterday morning; is sensible, and replies to questions put to him; pulse seventy, and soft; pupils somewhat more dilated, but they do not contract when the light is suddenly increased. The blindness, of which he complained yesterday, is gone off; he says that he can see. No return of the vomiting; occasional twitchings of the limbs, with great pain in the head. Ol. Ricini statim.

Twelve P.M.—Pulse sixty, full and hard; very restless; bowels moved twice. V.S. ad ℥xvi. After the bleeding the pulse increased ten beats in the minute, and became softer.

16th.—Slept well since the bleeding; is sensible, and appears to see; pupils inactive; great pain and soreness of the head; bowels moved twice in the night; gets out of bed to make water; pulse seventy-two, and weak; skin dry.

Pil. Hyd. Submur. c. Colocynth gr. x. hâc nocte. Mist. Salin. Cathart, mane.

17th.—A quiet night. Head very painful; pupils act slightly; tongue white; bowels moved three times.

Hirudines xx. occipiti. Pulv. Scam. c. Cal. gr. x.

Eight P.M.—Pulse seventy-four; pupils more sensible to light, but does not appear on the whole better: he moans much.

Hirudines xxx. occipiti.

18th.—Slept well until two A.M. when he was seized with a general convulsion, which returned several times before six o'clock. When he was seen after the convulsions had ceased, his pulse was 112 and small, countenance anxious, skin hot, tongue white, pupils sluggish; bowels freely opened.

From the date of this report he gradually improved.

---

II. *Slight Concussion of Brain, with venous Bleeding from the Ear.*

J. L. æt. sixty-one, admitted at St. Thomas's Hospital, under Mr. Green, November 6th. Is keeper of the gates at one of the bridges, and was attempting to stop a horse which had run away, by closing the gate, when the animal ran against him, and threw him down. He was taken up insensible, and vomited several times. When brought to the hospital, an hour after the accident, he was still insensible, but soon began to recover his consciousness, and in half an hour was able to answer questions. A lacerated wound, two inches in length, was discovered on the occiput, but no fracture was perceptible there. There was fracture of the radius and ulna of one arm. Bleeding from one ear was observed soon after his admission; and during the remainder of the day three or four ounces of venous blood slowly oozed out. The pulse, which was very weak on his admission, rose soon after; but the breathing continued laborious the whole evening, and he occasionally was delirious.

Mist. Cathart. Splints to the arm.

7th.—Slept little, but no bad symptoms.

8th.—Went out well, as far as the head was concerned.

---

### III. *Fracture of the Cranium, followed by Depositions of Lymph in the Lung and on the Pleura.*

A. B. aged forty-five, a distiller's collecting clerk, in the habit of drinking great quantities of spirits.

This patient was brought into St. Thomas's Hospital with a comminuted fracture of the patella, and a punctured wound on the forehead, which were stated to be the consequences of a fall from some height. The symptoms of injury to the brain or its coverings were so slight, that none was suspected: he was, however, bled to twelve ounces, as a measure of precaution.

The following day, his pulse being rather hard and quick, he was again bled, and some purgative administered.

There being no evidence to the contrary, he was believed to be doing well until November 19th, the fractured patella obtaining the chief share of attention in the interval: on that day, however, Mr. Travers found him complaining of pain in the head, and discovered that fungous granulations were sprouting up out of the wound on the forehead. He put in a probe, and felt the cranium denuded to some extent; and, having dilated the wound, he found that a portion of the cranium, containing nearly two square inches, was bare, indicating a separation of the dura mater from the bone. The only other symptom in addition to the wound in the head was a purulent discharge from one of the nostrils.

C. C. ad ℥xij. Rep^r Pil. purg.

Nov. 20th.—The patient being much worse, Dr. Williams saw him, and found him suffering from a violent pain in the right hypochondrium, similar to that produced by the passing of a gallstone, and unattended by any decisive symptoms of inflammation.

C. C. hypochondrio dextro.—Magnes. Sulph. ℥i. ex Mist. Camph. sextis horis.

21st.—Complains of pain in side, but none in the head; delirious.

22d.—No alteration, except that he is sensible, and, when asked where his pain is, complains of his side.

23d.—Died this day; delirious before death.

*Sectio cadaveris.*—The denuded portion of the cranium appeared roughened and bloodless; a very minute fissure, unobserved before death, extended vertically through it as far as the orbit. After the brain had been removed, a continuation of the same fissure was traced upon the orbitar plate of the frontal bone, where it soon became divided into two, one of which ran along the cribriform plate of the ethmoid bone, close to the crista galli, and terminated in the processus olivaris, which was broken into several portions; while the other, taking a direction outward for a short distance, again turned inward, and met the first near its termination. A considerable portion of the orbitar plate was thus detached, and slightly depressed upon the parts below; a portion of the cribriform plate was also separated. The internal surface of the frontal bone, where the fissure commenced, was, like the external, denuded of its periosteum; the dura mater roughened from absorption, and bloodless. Between it and the dura mater there was a small collection of pus; the membrane was softened, as if beginning to slough; and in the anterior lobe of the cerebrum behind it were seen what appeared to be the effects of acute inflammation following laceration, viz. a breaking-up of the cerebral substance, and conversion of a part of it into imperfect pus. Pus was also found lying on the cribriform plate over the fissure, and the dura mater there was sloughy: thus the discharge of pus through the nostril was accounted for. The cavity of the right pleura contained a pint or two of lemon-coloured transparent serum; the surface of the pleura covering the lung was covered with a layer of apparently recently effused lymph. Entering into the substance of the same lung, to the depth of about an inch, were several finger-shaped deposits of white and tolerably firm lymph: none of these had proceeded so far as to be softened or converted into pus. The left lung was healthy. The liver was very large, but healthy in its structure.

---

### IV. *Injury of the Head; Disease of the Bone.*

Thomas Price, a postboy, thirty-two or thirty-three years of age, was riding a vicious horse in the month of July, 1827, when the animal threw him with considerable force upon his head. He was stunned for some minutes by the fall, but no wound of the scalp was produced, and, after the immediate effects of the injury had passed, he was able to return to his usual employment. In the course of a few weeks there came on such pain in the forehead that he could not endure the exertion of riding. Some months had now elapsed since the infliction of the injury, when an abscess formed upon the vertex, and was opened at the commencement of the present year.

On admission into St. George's Hospital, the 8th of last October, there was constant, often violent, pain in the forehead, giddiness, and indistinct vision. The iris of both eyes was very irregular in its action, but the pupil of the right was mostly dilated, that of the left being natural in size. Near the crown of the head was a small and nearly circular ulcerated opening of the soft parts, through which the probe passed down to exposed and carious bone. The man was emaciated; his aspect pallid, his expression somewhat vacant. The tongue was clean; the pulse was always quick. He said that his memory was impaired.

On the 10th, Mr. BRODIE enlarged the opening leading to the bone, but found the diseased portion too firm to be removed by any thing short of the trephine. Accordingly, on the 16th, having previously obtained the advice and concurrence of Mr. KEATE, a crucial incision was made in the scalp, and a small trephine applied at the spot where the dead bone was felt. The portion embraced within the circle of the instrument having been removed, and a very copious bleeding, which occurred from the scalp and substance of the bone, being checked by pressure, the dura mater was examined, and found to present on its surface a little coagulable lymph, without any purulent matter. On inspecting the bone, the caries was seen to have stopped at the diploë, the tabula vitrea remaining sound. Two or three of the bleeding vessels were secured, and the flaps of the scalp brought together with a suture.

No unfavorable symptoms followed the operation, nor was it succeeded by any very marked relief. Some œdematous swelling took place about the occiput, and he suffered, on the 17th, from headache and soreness of the scalp. The pulse at this time was 100; the tongue clean and moist; the bowels not open since the operation. He was bled to twelve ounces, and ordered

**Haustus Sennæ ℥ij. statim. Haust. Salin. c. Vin. Ant. Tart. m.xv. M. sextis horis.**

He vomited that night, and again on the 18th, when, however, he was better. The pulse was 124, the tongue moist, the bowels open; and his head, he said, was easier than it had been for months. Six ounces only of blood had been taken, and that was not buffed.

**Rep^r H. Salin. sine Liq. Ant. Tart.—Cucurbit. cruent. inter scapulas. V.S. ad ℥x.**

Next day the pain of the head was worse; the pulse 108, tongue moist, the right pupil more dilated than the left. By the 20th, the pain had entirely disappeared; the tongue was clean, the pulse ranging from 100 to 108.

Thus he continued, with very little change, till the 18th November. On the 17th, he had taken a dose of Haustus Sennæ, and next day he was seized with sickness, succeeded by headache and pain in the forehead, infinitely worse than ever it had been before. The pulse, which for some days had been as low as eighty, rose and was fuller. The wound had nearly closed, but a free passage still remained for the exit of matter.

**Cuc. cr. nuch. ad ℥x.—H. Sal. ℥iss.; Magn. Sulph. ʒi. sextis horis.**

On the 19th, being no better, he was bled once more to ten ounces, and a blister applied to the neck. He vomited several times that evening, and the 20th brought no relief, at least to the pain in the head. On the 21st, the pain in the forehead was excruciating, and the head was seen to move at every pulsation of the heart. The pulse was ninety, and full; the thirst was great; the countenance anxious, and depressed in the highest degree. At one time the patient would be calm; in a very few minutes rolling in his bed in absolute agony.

Small bleedings were repeated on the 22d and 23d, but the blood was buffed on one occasion only.

On the 24th, the termination of the case was perceptibly approaching. The pain was dreadful, the pulse very weak, the tongue becoming brown, the manner even more than half delirious. An increased discharge had taken place from the wound in the night, and today Messrs. Brodie and Keate divided the cicatrix, and freely enlarged the small opening that remained. No matter, however, was found or escaped.

Pulv. Ipec. C. gr. x.; H. Salin. statim, et h. s. repetend. si opus sit.

The 25th brought no relief whatever. The tongue was brown and dry, the pulse quick and feeble, and something like brain mixed up in coagulum was found upon the wound. In this condition the patient was removed from the house by his friends, to a distance of one or two miles, presenting a most melancholy spectacle. He died that evening. No dissection was permitted; but hernia cerebri had taken place.

---

